# Gut Metagenome Reveals the Microbiome Signatures in Tibetan and Black Pigs

**DOI:** 10.3390/ani15050753

**Published:** 2025-03-06

**Authors:** Xue Bai, Yiren Gu, Diyan Li, Mingzhou Li

**Affiliations:** 1 State Key Laboratory of Swine and Poultry Breeding Industry, College of Animal Science and Technology, Sichuan Agricultural University, Chengdu 611130, China; 18792972819@163.com; 2 College of Animal and Veterinary Sciences, Southwest Minzu University, Chengdu 610041, China; guyiren1128@163.com; 3 School of Pharmacy, Chengdu University, Chengdu 610106, China

**Keywords:** Tibetan pig, black pig, metagenome, gastrointestinal tract, composition, function

## Abstract

The harsh conditions of the Qinghai–Tibet Plateau pose significant physiological challenges to local fauna, often resulting in gastrointestinal disorders. However, Tibetan pigs have exhibited remarkable adaptability to the high-altitude stress of the Tibetan Plateau, a phenomenon that remains not fully understood in terms of their gastrointestinal microbiota. This study collected 57 gastrointestinal tract samples from Tibetan pigs (*n* = 6) and plain black pigs (*n* = 6) with comparable genetic backgrounds. Samples from the stomach, jejunum, cecum, colon, and rectum, underwent comprehensive metagenomic analysis to elucidate the gut microbiota-related adaptive mechanisms in Tibetan pigs to the extreme high-altitude environment. A predominance of *Pseudomonadota* was observed within gut microbiome of Tibetan pigs. Significant differences in the microbial composition were also identified across the tested gastrointestinal segments, with 18 genera and 141 species exhibiting differential abundance. Genera such as *Bifidobacterium*, *Megasphaera*, *Fusobacterium*, and *Mitsuokella* were significantly more abundant in Tibetan pigs than in their lowland counterparts, suggesting specialized adaptations. Network analysis found greater complexity and modularity in the microbiota of Tibetan pigs compared to black pigs, indicating enhanced ecological stability and adaptability. Functional analysis revealed that the Tibetan pig microbiota was particularly enriched with bacterial species involved in metabolic pathways for propionate and butyrate, key short-chain fatty acids that support energy provision under low-oxygen conditions. The enzymatic profiles of Tibetan pigs, characterized by elevated levels of 4-hydroxybutyrate dehydrogenase and glutaconyl-CoA decarboxylase, highlighted a robust fatty acid metabolism and enhanced tricarboxylic acid cycle activity. In contrast, the gut microbiome of plain black pigs showed a reliance on the succinate pathway, with a reduced butyrate metabolism and lower metabolic flexibility. Taken together, these results demonstrate the crucial role of the gastrointestinal microbiota in the adaptation of Tibetan pigs to high-altitude environments by optimizing carbohydrate metabolism and short-chain fatty acid production for efficient energy utilization. This study not only highlights the metabolic benefits conferred by the gut microbiota of Tibetan pigs in extreme environments, but also advances our understanding of the adaptive gastrointestinal mechanisms in plateau-dwelling animals. These insights lay the foundation for exploring metabolic interventions to support health and performance in high-altitude conditions.

## Simple Summary

The harsh Qinghai–Tibet Plateau conditions pose physiological challenges to local fauna, often causing gastrointestinal issues. Tibetan pigs, however, show remarkable adaptability to this high-altitude stress. This study analyzed 57 gastrointestinal samples from Tibetan pigs and genetically similar plain black pigs, focusing on the gut microbiota’s role in adaptation. Metagenomic analysis revealed a predominance of *Bacillota* and *Lactobacillus* in Tibetan pigs, with significant differences in microbial composition including more abundant *Bifidobacterium*, *Megasphaera*, *Fusobacterium*, and *Mitsuokella* compared to lowland pigs. Network analysis indicated greater microbiota complexity and stability in Tibetan pigs, with functional analysis showing enrichment in metabolic pathways for key short-chain fatty acids like propionate and butyrate, enhancing energy provision under low-oxygen conditions. These findings advance our understanding of gastrointestinal adaptations in high-altitude dwelling animals.

## 1. Introduction

Tibetan pigs (*Sus scrofa domesticus*) are a unique indigenous breed native to the Qinghai–Tibet Plateau, a region characterized by extreme environmental conditions including hypoxia, severe cold, intense ultraviolet radiation (UVR), and limited food resources [1,2]. Over centuries of natural and artificial selection, Tibetan pigs have developed distinct genetic and physiological traits that enable them to thrive in high-altitude environments [3]. These adaptations not only highlight the remarkable plasticity of this unique breed but also provide a valuable genetic resource for understanding mechanisms of high-altitude resilience, particularly in response to oxygen deprivation and environmental stressors.

The gastrointestinal (GI) tract is the main organ system for nutrient absorption and energy metabolism [4,5,6]. Its physiology in pigs closely resembles that of humans, making pigs a valuable model for studying gut-related metabolic processes [7]. The GI tract is responsible for the breakdown of food [8], nutrient absorption, and hosting [9] a rich microbial community that plays a pivotal role [10,11] in fermenting complex carbohydrates into short-chain fatty acids (SCFAs) such as acetate, propionate, and butyrate [12,13,14]. These SCFAs are essential for supporting host energy metabolism and maintaining overall health. Given the functional and physiological parallels between pigs and humans, understanding the gut microbiota’s role in pigs can provide insights into human health and adaptation to extreme environments.

Gut microbiota have emerged as a key regulator of host biology, influencing processes such as nutrient production and energy homeostasis [15,16]. The composition and diversity of the gut microbiota are closely linked to host health and environmental adaptation [17]. Notably, high-altitude environments are associated with significant changes in gut microbiota composition and diversity, reflecting the host’s ability to adapt to extreme conditions [18,19]. Animals living at high altitudes show an increase in the relative abundance of *Bacillota* [20,21]. This shift is thought to enhance energy-harvesting efficiency, helping to compensate for the increased energy demands imposed by low oxygen and cold temperatures. Additionally, certain bacterial taxa, such as *Lactobacillus*, was considered probiotic [22], and has been shown to promote gut functionality and restore microbial balance, thereby enhancing host adaptability to high-altitude environments [23].

Tibetan pigs, as a high-altitude-adapted breed, offer a unique model for studying the interplay between host physiology and gut microbiota in extreme environments. Previous studies that primarily focused on characterizing the fecal microbiota of Tibetan pigs revealed enrichment in bacterial taxa such as *Acinetobacter*, *Pseudomonas*, and *Sphingobacterium* [24]. The fecal microbiome of Tibetan pigs is also enriched in members of *Fibrobacterota* and *Elusimicrobia*, which are actively involved in the production of SCFAs, medium-chain fatty acids, essential amino acids, B vitamins, and cofactors that support host energy metabolism and adaptation to hypoxia and cold stress [25,26]. Despite these findings, there is a lack of research exploring the gut microbiota composition across different intestinal segments and their specific roles in high-altitude adaptation.

To address this gap in knowledge, we performed a comparative metagenomic study of five GI tract segments (stomach, jejunum, cecum, colon, and rectum) from Tibetan pigs and low-altitude black pigs. By comparing the microbial composition and functional potential of these regions in the two porcine species, we aim to elucidate the role of gut microbiota in supporting high-altitude adaptation and to identify metabolic pathways and microbial taxa that contribute to host survival in extreme environments. This study provides a comprehensive understanding of the gut microbiota’s involvement in the environmental adaptation of the host, and it lays the foundation for exploring microbial-based strategies to improve animal and human health in challenging conditions.

## 2. Materials and Methods

### 2.1. Animal Management and Sample Collection

In this study, we selected six Sichuan local black pigs (originating from Suining, Sichuan, China, located at 30°53′ N, 105°59′ E, with an altitude of 461 m) and six Tibetan fragrant pigs (from Daocheng, Sichuan, China, situated at 28°26′ N, 99°86′ E, at an altitude of 3750 m). Following a stable rearing period of 300 days, all the pigs were transported to a nearby commercial slaughterhouse. In this study, all experimental pigs did not have diarrhea within one month before sampling, and no antibiotics were used during the entire experimental period. The basic growth data of experimental pigs are shown in Table S1. In accordance with standard animal sampling protocols, the pigs were deprived of food for 24 h and water for 2 h prior to slaughter. When collecting samples, we separated the various sections of the GI tract and collected contents from the stomach, jejunum, cecum, colon, and rectum. Immediately after collection, all samples were rapidly frozen in liquid nitrogen, temporarily stored in dry ice, and then transported to the laboratory for storage at −80 °C. For each type of pig, we obtained six biological replicates and three technical replicates.

### 2.2. Library Preparation and Sequencing

High-quality total DNA was extracted from the collected samples using a QIAGEN DNeasy PowerSoil Kit (Qiagen, Gaithersburg, MD, USA). The DNA concentration was measured using a NanoDrop spectrophotometer and further verified with the Qubit^®^ dsDNA Assay Kit in a Qubit^®^ 2.0 Flurometer (Life Technologies, Carlsbad, CA, USA), ensuring the OD value was between 1.8 and 2.0 and the DNA content was above 1 ug for library construction.

The Illumina TruSeq DNA PCR-Free Sample Preparation Kit (Illumina, San Diego, CA, USA) was utilized for library construction, which included DNA fragmentation, end repair, adapter ligation, and purification and enrichment with magnetic beads to avoid PCR amplification bias and accurately reflect the sample’s microbial community structure [27]. The constructed libraries were quality-assessed by checking fragment size distribution with an Agilent 2100 Bioanalyzer (Agilent, Santa Clara, CA, USA) and precisely measuring concentration with a Qubit 2.0 [28] fluorometer. High-throughput sequencing was conducted on the Illumina NovaSeq 6000 platform in a 2 × 150 bp paired-end mode, and the sequencing service was provided by BGI (Shenzhen, China).

### 2.3. Sequence Processing, Assembly, and Quality Assessment in Metagenomics

Metagenomic data underwent stringent preprocessing to ensure data quality. Sequences were subjected to quality control using Trimmomatic version 0.38, where reads with a Phred score below 15 across a 4-base sliding window and those shorter than 50 bp were discarded, and sequencing adapters were eliminated^4^. To deplete host-derived sequences, quality-filtered reads were aligned to the Sus scrofa reference genome (Sscrofa 11.1) using Bowtie2 (version 2.4.2-15). MEGAHIT (version 1.29.0) was employed to conduct both co-assembly and individual assembly of the host-depleted metagenomic datasets. Prior to co-assembly, the normalization of all metagenomic samples was carried out using BBNorm from the BBTools suite (version 38.796). Post-assembly, sequences from each sample were mapped back to their respective assemblies; for the co-assembled dataset, Bowtie2 was used for mapping, while for individual assemblies, each sample was aligned to its own assembly output.

### 2.4. Gene Taxonomy and Functional Annotation

The protein sequences from the unique gene catalog were aligned against the NR database via Diamond [29] (version 0.8.35, e-values ≤ 1 × 10^−5^) to acquire taxonomic classifications. Similarly, the annotation of the COG (Cluster of Orthologous Groups of Proteins) for selected sequences was executed using Diamond [29] (version 0.8.35, e-values ≤ 1 × 10^−5^) and aimed at the eggNOG database. Additionally, the functional mapping to the KEGG (Kyoto Encyclopedia of Genes and Genomes) pathways was performed with the same version of Diamond [29], adhering to the same e-value threshold.

### 2.5. Alpha and Beta Diversity

Alpha diversity was calculated to reflect the variety of species within a specific area or ecosystem. This metric was utilized to assess the complexity and richness of species in samples, with the Shannon index being employed to determine community diversity levels. Variations in the alpha diversity index among the four groups were analyzed using the Wilcoxon rank sum test in R software (version 4.3.2). In addition, principal coordinate analysis (PCoA) was performed on the relative abundance of bacterial genus level and KEGG functional annotation. This analysis utilized the vegan package in R (version 4.3.2) for graphical representation.

## 3. Results

### 3.1. Metagenomic Sequencing Results Statistics

In this study, metagenomic sequencing was performed on 57 samples of the stomach, jejunum, cecum, colon, and rectum of Tibetan pigs (*n* = 6) and black pigs (*n* = 6). After removing the adapters and low-quality sequences, a total of 685.10 GB (Mean 12.02 GB) clean bases and 2,283,671,087 reads were obtained. The Q20 index was above 98%, and the Q30 was above 91%; the average percentage of G/C bases to the total number of bases was 43.23%. After removing the host-mapping reads (pig gene data), a total of 2,014,799,513 non-porcine reads were obtained, totaling 297.24 GB (Mean 5.21 GB) of clean data (Table S2). Non-redundant data were obtained after gene prediction and de-redundancy, and catalog genes were constructed.

### 3.2. Composition Characteristics of GI Flora in Tibetan Pigs and Black Pigs

**Figure 1:**
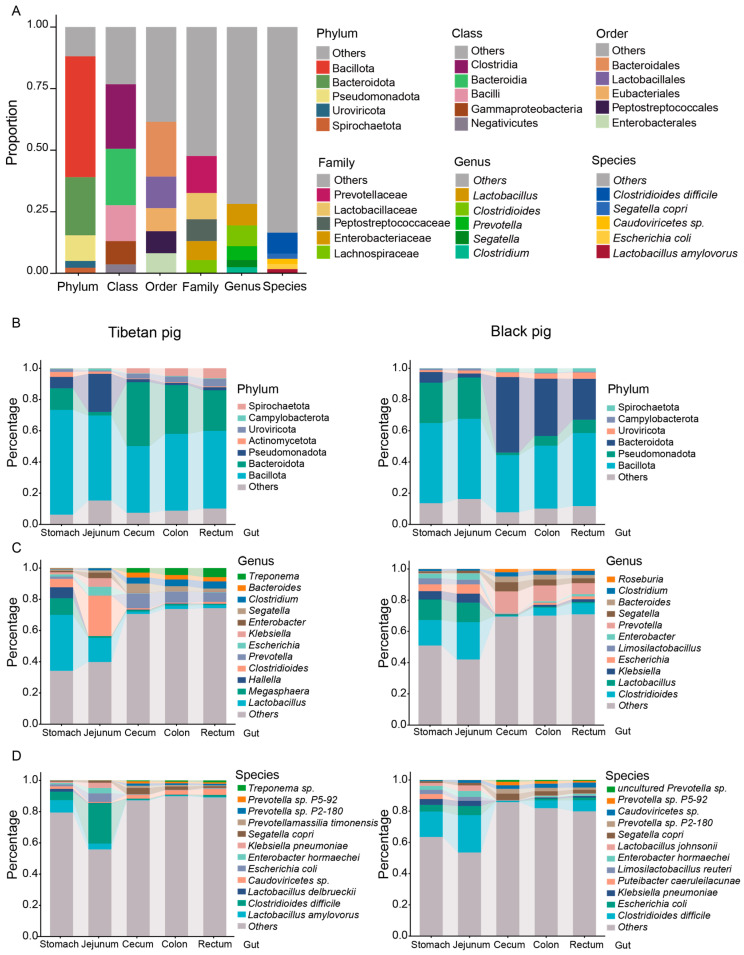
The intestinal microbial composition of Tibetan pigs and plain black pigs. (**A**) The overall composition of the top five relative abundances of Tibetan pigs and black pigs at the phylum, class, order, family, genus, and species level. (**B**–**D**) The composition of the top five relative abundances of microorganisms in different intestinal segments of Tibetan pigs and black pigs at the phylum (**B**), genus (**C**), and species (**D**) level.

After comparing the non-redundant gene set with the NR database, the species were annotated. In the GI microbiota of all pigs, phyla *Bacillota*, *Bacteroidota*, *Pseudomonadota*, *Caudoviricetes*, and *Spirochaetota* dominated in various intestinal sites. Among them, the relative abundance of *Bacillota* was 49.1%, *Bacteroidetes* was 23.6%, *Pseudomonas* was 10.4%, *Caudoviricetes* and *Spirochaetota* were 2.7% and 2.3%, respectively, and the abundance of the remaining phyla was less than 2% (Figure 1A, Table S3). *Bacillota* and *Bacteroidetes* are known to be important in the fermentation of complex carbohydrates and the production of short-chain fatty acids.

The predominant genera identified in the porcine GI included *Lactobacillus* (8.7%), *Clostridioides* (8.5%), *Prevotella* (5.6%), *Segatella* (2.9%), and *Clostridium* (2.5%). At the species level, *Clostridioides difficile* (8.5%), *Segatella copri* (2.2%), *Caudoviricetes* sp. (2.1%), *Escherichia coli* (2.0%), and *Lactobacillus amylovorus* (*L. amylovorus*, 1.7%) were the most abundant (Figure 1A, Table S3).

At the phylum level, the gut microbial composition of Tibetan pigs and black pigs showed significant differences across different GI tract sites. *Bacillota* and *Pseudomonas* were more abundant in the stomach and jejunum of both pig breeds, whereas they were significantly reduced in the hindgut segments (cecum, colon, and rectum). Furthermore, there was a significant difference in the relative abundance of *Pseudomonadota* between Tibetan pigs and black pigs, with *Bacillota* being more abundant in Tibetan pigs and *Bacteroidota* being more abundant in black pigs (Figure S2). *Bacteroidetes* was most prevalent in the cecum, colon, and rectum, and least abundant in the stomach and jejunum (Figure 1B). This distribution was consistent in both pig breeds, suggesting the role of *Bacteroidetes* in fiber degradation and metabolism in the hindgut segment.

At the genus level, the gut microbial composition of Tibetan pigs and black pigs also showed significant differences: the relative abundance of *Lactobacillus* and *Megasphaera* was significantly higher in the stomach and jejunum of Tibetan pigs, suggesting that these genera may be involved in the initial fermentation and metabolism of the high-fiber diet (Figure 1C). In contrast, *Clostridium difficile* was significantly higher in the stomach and jejunum of black pigs, suggesting that the microbiota structure of these animals may be more adapted to a low-fiber, high-energy diet. In the hindgut segment, the abundance of *Roseburia* was significantly increased in black pigs, indicating a role in the synthesis of short-chain fatty acids.

At the species level, *Clostridium difficile* was the predominant bacterial species in the stomach and jejunum of both Tibetan pigs and black pigs (Figure 1D), pointing at the implication of this species in the initial fermentation and metabolism in the stomach and jejunum.

**Figure 2:**
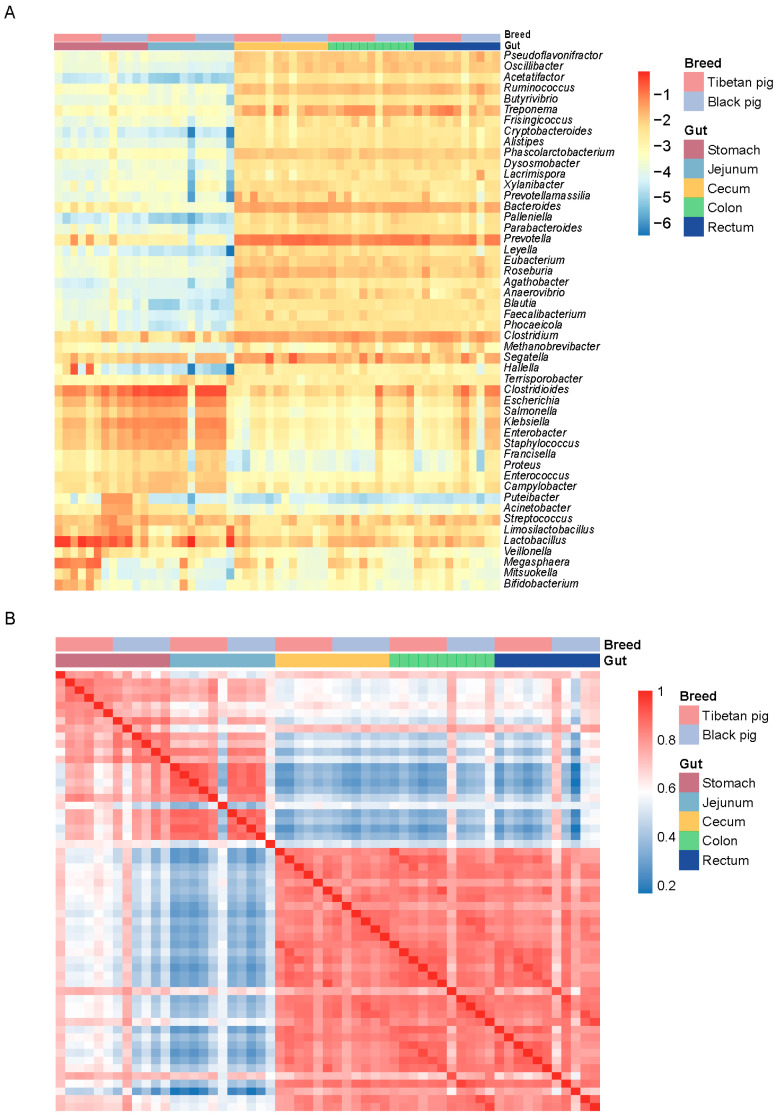
Distribution of GI microorganisms in Tibetan pigs and plain black pigs across each intestinal segment. (**A**) Distribution of the top 50 bacterial genera with the highest relative abundance in each GI segment. (**B**) Correlation heat map at the species level.

To further analyze the distribution characteristics of differential bacterial communities, we created a heat map of the top 50 bacterial genera by relative abundance. The results showed that although there was no significant difference in the overall digestive tract microbial composition between Tibetan pigs and black pigs, there were significant differences in the relative abundance of certain bacterial genera. The relative abundances of *Lactobacillus* and *Megasphaera* were significantly higher in Tibetan pigs than in black pigs, whereas the abundances of *Escherichia* and *Clostridium difficile* were higher in black pigs. In the hindgut, Tibetan pigs were more enriched with bacteria related to fiber degradation such as *Ruminococcus* and *Prevotella*, while black pigs had more potential pathogenic bacteria such as *Klebsiella* and *Salmonella*, with the genus *Salmonella* being more abundant (Figure 2A). A species-level correlation analysis further revealed the distinct clustering characteristics of GI microorganisms across different intestinal segments in Tibetan and black pigs. The six replicate samples from the same intestinal segment showed the highest correlation, indicating a highly consistent distribution pattern of GI bacteria within the local environment (Figure 2B).

### 3.3. Diversity of GIl Flora of Tibetan Pigs and Black Pigs

**Figure 3:**
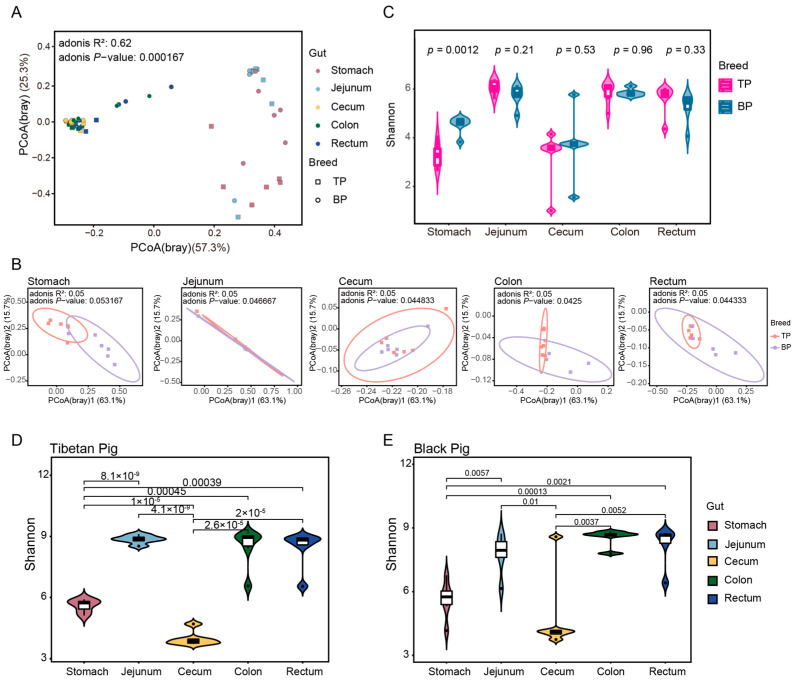
Diversity of intestinal microbial communities of Tibetan pigs and plain black pigs. (**A**,**B**) PCoA of samples based on Bray–Curtis distance at the genus levels. (**C**–**E**) Alpha diversity comparison based on Shannon diversity indices at the genus levels.

A principal component analysis (PCoA) based on Bray–Curtis distance revealed a clear clustering of the GI microbiota across different intestinal segments and between Tibetan and black pigs (Figure 3A,B). The distances between samples collected from the hindgut segments (cecum, colon, and rectum) were smaller, resulting in tighter clustering, whereas the microbial composition of the stomach and jejunum differed significantly from that of the hindgut segments. The Adonis test was further used for verification, corroborating PCoA findings and showing significant differences in the bacterial composition of the GI flora at both the genus and the species levels (genus level: R^2^ = 0.62, *p* < 0.001; species level: R^2^ = 0.56, *p* < 0.001). These results provide evidence of significant differences in the overall structure of the GI microbiota between high-altitude (Tibetan) and plain (black) pigs, with distinct regional distribution of microorganisms across different GI segments. The observed regional distribution may be closely related to the differences in physiological functions and nutritional matrices of different intestinal segments.

In the alpha diversity analysis, significant differences between pig breeds were only observed in the stomach samples, where Tibetan pigs exhibited a significantly higher Shannon diversity index compared to black pigs (*t*-test, *p* = 0.0012, Figure 3C). This stomach-specific difference may be linked to variations in the dietary structure and initial digestive process of the two pig breeds. The alpha diversity of Tibetan pigs among different intestinal sites was greater than that of black pigs (Figure 3D,E).

### 3.4. Differences in GI Flora Between Tibetan Pigs and Black Pigs

**Figure 4:**
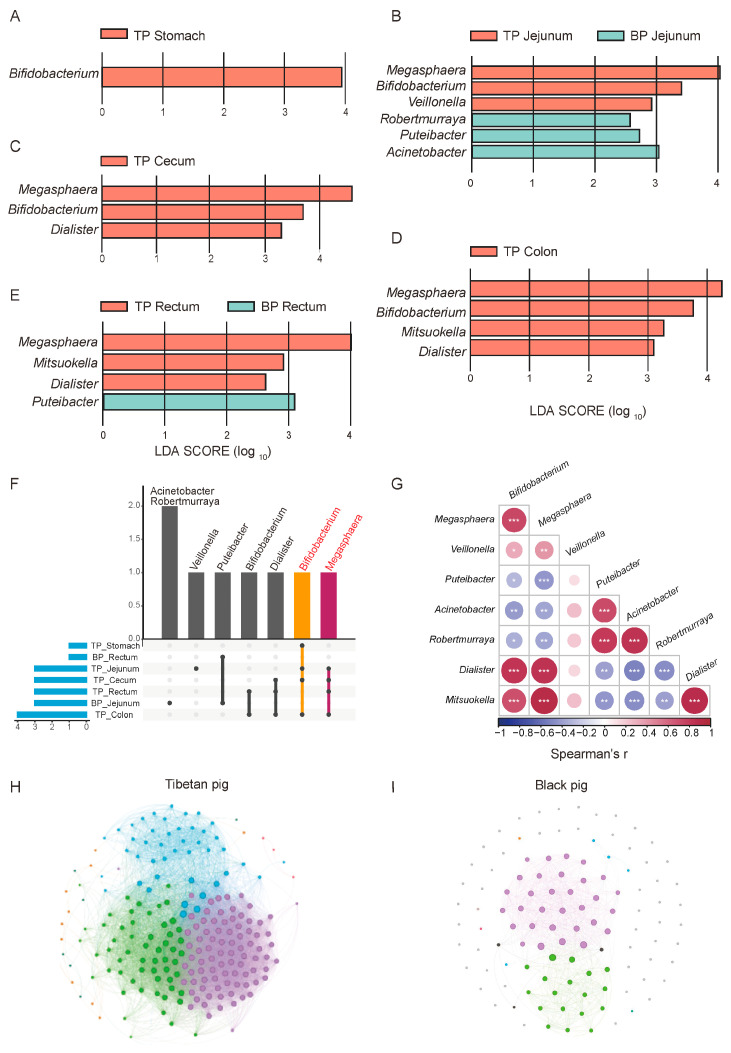
Differential analysis of GI flora between Tibetan pigs and common black pigs. (**A**–**E**) LEfSe differential maps at the genus level across different GI segments in Tibetan pigs and black pigs. (**F**) Upset map of the distribution of different bacterial genera in each part. (**G**) Spearman correlation heat map of different bacterial genera, * *p*  <  0.05, ** *p*  <  0.01, and *** *p*  <  0.001. Correlation network diagrams of the GI microbiome in Tibetan pigs (**H**) and black pigs (**I**) based on the relative abundance of bacterial genera.

Potential differences in the composition of the GI flora between high-altitude Tibetan pigs and low-altitude black pigs were assessed with the LEfSe test (*p* < 0.05, LDA > 2), comparing the microbiota across different GI sites at the genus and species levels (relative abundance > 0.1%). A total of 18 genera and 132 species of microorganisms significantly differed between the different GI parts of high-altitude and low-altitude pigs, including *Bifidobacterium*, *Megasphaera*, *Verticillium*, *Veillonella*, *Puteibacter*, *Acinetobacter*, *Robertmurraya*, *Dialister*, and *Mitsuokella*. In fact, 14 of the 18 bacterial genera were found to be significantly more abundant in high-altitude Tibetan pigs (*p* < 0.05, Figure 4A–D). Among these genera, *Bifidobacterium*, *Megasphaera*, *Dialister*, and *Mitsuokana* were mainly enriched in the stomach, jejunum, cecum, and colon, while *Mitsuokella* was significantly more abundant in the jejunum, cecum, colon, and rectum (Figure 4F). These enriched genera are involved in the metabolism of high-fiber diets, breaking down fiber and producing SCFAs, such as acetate, propionate, and butyrate, to provide energy for the host and facilitate adaptation to high-altitude environments. In contrast, the jejunum and rectum of plain black pigs were enriched with *Puteibacter*, *Acinetobacter*, and *Robertmurraya*, reflecting the GI microbiome adaptation to low-altitude environments.

We next explored the synergistic role of microorganisms in adaptation to high-altitude environments. A correlation analysis revealed significant positive correlations between the bacterial genera were significantly enriched in high-altitude Tibetan pigs (such as *Bifidobacterium*, *Megasphaera*, *Fusobacterium*, and *Misuoka*), suggesting their synergistic participation in metabolic processes like carbohydrate fermentation and fiber decomposition (Figure 4G). Additionally, these enriched bacterial genera exhibited a negative correlation with genera which were significantly more abundant in plain black pigs, such as *Pseudomonas* and *Acinetobacter*. This mutually exclusive pattern reflects differences in the functional microbial characteristics and metabolic strategies between the two pig breeds in different altitude environments.

To further elucidate the ecological significance of this microbial synergy, we compared the intestinal microbial community structure of high-altitude Tibetan pigs and plain black pigs through a genus-level correlation network analysis. Overall, the complexity of the GI microbial network in Tibetan pigs is significantly higher than that in plain black pigs. The nodes in the Tibetan pigs’ network are more strongly correlated, present an obvious modular structure, and are enriched with core genera (Figure 4H,I). This reflects the higher collaboration and functional differentiation of the GI microbial communities of Tibetan pigs, which may help in their adaptation to the low-oxygen and low-temperature conditions of high-altitude environments by enhancing ecological stability.

In contrast, the microbial network structure of plain black pigs is relatively simple, with a lower degree of modularity, and weaker collaboration between bacterial communities. This difference suggests that the GI microbiota of plain black pigs is under less adaptive pressure in the relatively stable low-altitude environment, with bacterial community functions primarily focused on meeting basic metabolic needs, while lacking the ability to respond to complex environmental conditions. In summary, the GI microbiome of high-altitude Tibetan pigs forms an ecological network with high modularity and functional differentiation, driven by synergistic effects, providing important metabolic support for their adaptation to the plateau environment.

**Figure 5:**
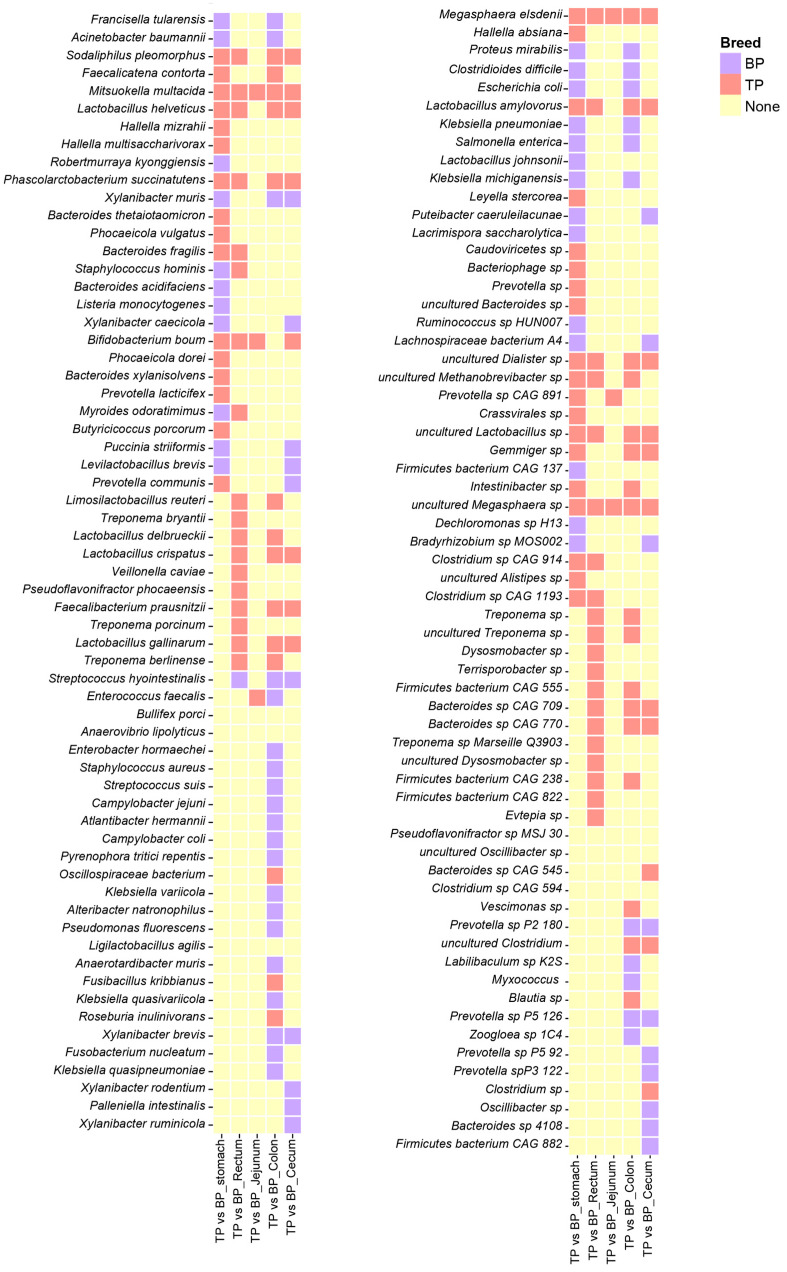
Differential analysis of the GI flora in Tibetan pigs and black pigs. Heat map showing LEfSe differential results at the species level across different parts of the GI tract in Tibetan and black pigs (LDA > 2, *p* < 0.05). The coral-red blocks indicate the enrichment of Tibetan pig species, the purple blocks indicate the enrichment of black pig species, and the yellow blocks indicate that neither Tibetan pig nor black pig showed enrichment characteristics in the corresponding parts.

The species correlation network analysis found that *Megasphaera elsdenii* (*M. elsdenii*) and *Mitsuokella multacida* (*M. multacida*) were significantly enriched across various intestinal segments of the Tibetan pigs, while *Bifidobacterium boum* was primarily distributed in the stomach, jejunum, cecum, and rectum. Additionally, *Clostridium* sp., *L. amylovorus*, *Phascolarctobacterium succinatutens*, and *Sodaliphilus pleomorphus* (*S. pleomorphus*) showed to be significantly enriched in the stomach, cecum, colon, and jejunum (Figure 5). The distribution of these bacterial species indicates that they may have specific functional roles and ecological adaptations in different GI segments.

### 3.5. KEGG Functional Enrichment Analysis

**Figure 6:**
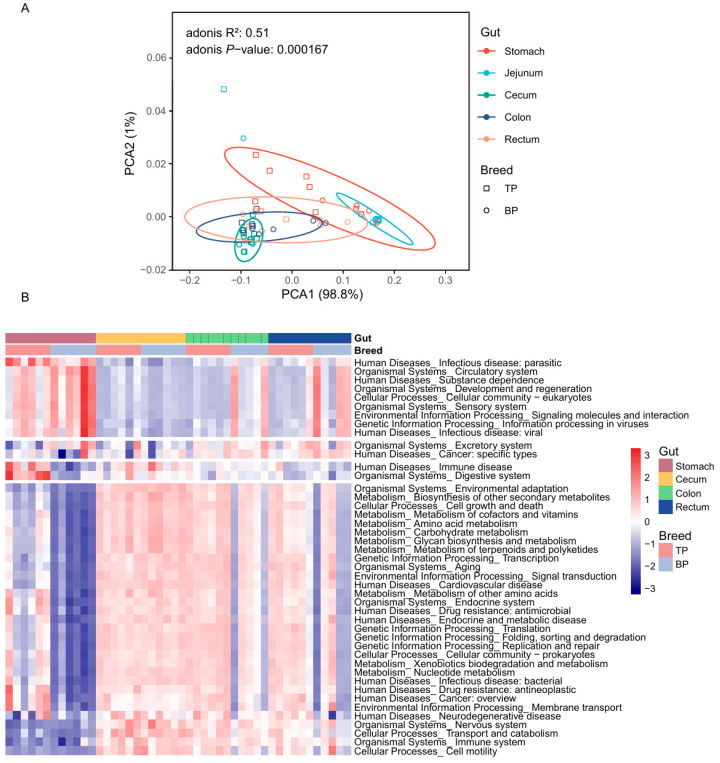
Functional differences in the GI microbiota of Tibetan pigs and plain black pigs. (**A**) PCoA diagram of functional genes in the GI microbiota of Tibetan and black pigs based on Bray–Curtis distance. (**B**) Heat map of differential functions of the GI microbiota based on KEGG pathway enrichment and LEfSe analysis in Tibetan and plain black pigs.

We annotated the functional genes of the GI flora in both pig breeds based on the egNOG database and obtained 18,579 gene annotations. Based on the Bray–Curtis distance, the annotated genes formed distinct clusters in the different parts of the GI tract of high-altitude Tibetan pigs and plain black pigs (Adonis test, R^2^ = 0.51, *p* < 0.001, Figure 6A and Figure S2). The distance between the functional groups of the stomach and jejunum was smaller, while the functional composition of the cecum, colon, and rectum was more similar. The sKEGG functional pathway enrichment analysis was performed, identifying 458 KEGG ontology (KO) pathways, in which ABC transporters (ko02010; mean ± standard deviation, 3.30 ± 1.33%), ribosomal genes (ko03010; 2.95 ± 1.02%), purine metabolism related genes (ko00230; 2.48 ± 0.78%), amino sugar and nucleotide sugar metabolic genes (ko00520; 1.93 ± 0.72%), and two-component systems (ko02020; 1.84 ± 0.71%) showed the highest enrichment rates (Figure 6B).

Analysis of the spatial distribution patterns of the functional composition of the GI flora in high-altitude Tibetan pigs and plain black pigs revealed significant differences in their functional performance across different intestinal sites (Figure 6B, Table S4). In the stomach of Tibetan pigs, microbial functions related to carbohydrate metabolism (i.e., starch/sucrose [ko00500] and amino/nucleotide sugar [ko00520] metabolism), amino acid metabolism (i.e., alanine, aspartate and glutamate metabolism [ko00250], cysteine and methionine metabolism [ko00270]), and secondary metabolites biosynthesis (i.e., monobactam biosynthesis [ko00261]) were significantly enriched, reflecting the stomach’s role in the initial breakdown of nutrients. In contrast, the microbial functions in the colon and rectum of Tibetan pigs were primarily associated with genetic information processing, with enriched pathways in replication and repair (ko03410) and RNA degradation (ko03018). This suggests that the microbiome of the posterior GI tract may support host cell renewal and genome stability by enhancing nucleic acid metabolism.

**Figure 7:**
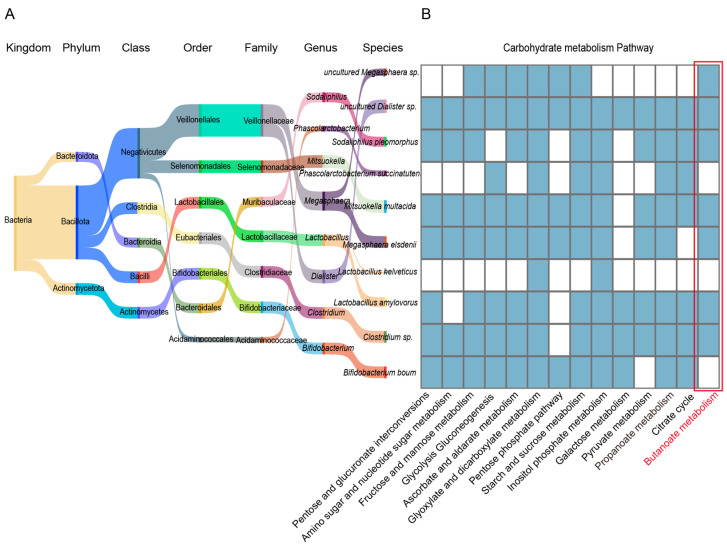
Functional analysis of bacterial strains in the GI tract of Tibetan pigs. (**A**) Sangkey diagram of bacterial species significantly enriched in 4–5 GI sites in Tibetan pigs. The name of the bacterial colony and the color of the corresponding flow direction represent the affiliation relationship. (**B**) Heat map of carbohydrate metabolism in the selected differential bacterial species: blue indicates high enrichment of the bacterial species in the corresponding pathway, while white indicates no enrichment.

To further clarify the functional contribution of differential bacterial species, we conducted an in-depth analysis, which revealed that the bacterial species enriched in specific parts of the Tibetan pig GI tract play key roles in the carbohydrate metabolic pathway, being widely involved in pentose and glucuronate interconversions (ko00040), amino sugar and nucleotide sugar metabolism (ko00520), fructose and mannose metabolism (ko00051), starch and sucrose metabolism (ko00500), and galactose metabolism (ko00052). In addition, we found that *Clostridium* sp., *L. amylovorus*, *M. elsdenii*, *M. multacida*, *S. pleomorphus*, uncultured *Dialister* sp., and uncultured *Megasphaera* sp. were significantly enriched in the propanoate (ko00640) and butanoate (ko00650) metabolic pathways (Figure 7A,B). They provide energy to the host by producing sSCFAs (such as propionate and butanoate), thus, supporting the energy metabolism required in high-altitude environments. Taken together, these results indicate that the GI microbiome of Tibetan pigs efficiently degrades and utilizes complex carbon sources (such as cellulose, galactose, and fructose) to provide energy to the host, and plays a key role in regulating host energy metabolism in the adaptation to high-altitude environments.

**Figure 8:**
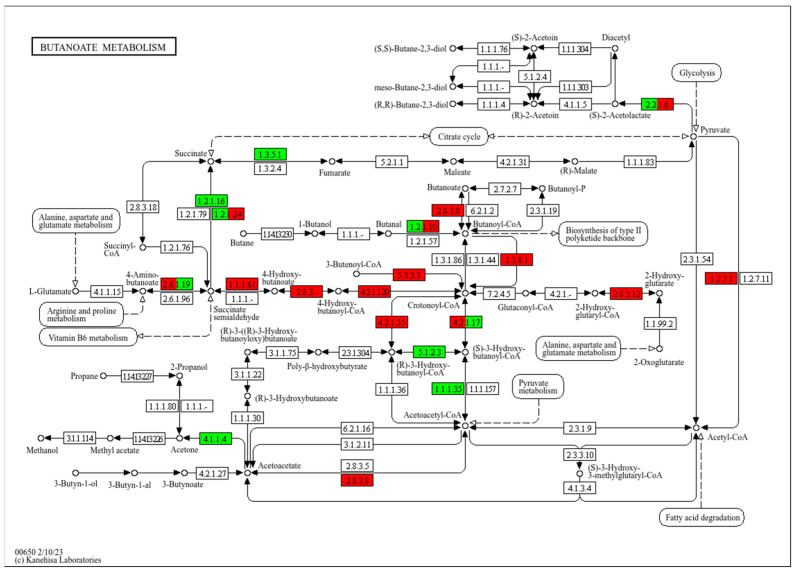
Schematic diagram of the main modules involved in the butyrate metabolic pathway (ko00650). Red indicates significantly higher relative abundance in Tibetan pigs, and green indicates significantly higher abundance in black pigs. Arrows in the figure represent molecular interactions or relationships; circles represent compounds, DNA, and other molecules; rectangles represent gene products (proteins, RNA); and rounded rectangles represent signal pathways.

To reveal the relationship between butyrate and energy metabolism, the core reaction network of butyrate metabolism, involving short-chain fatty acid metabolism, energy generation, and carbon source utilization, was analyzed in Tibetan pigs. The results showed an upregulation in the expression of key enzymes involved in butyrate metabolism, such as 4-hydroxybutyrate dehydrogenase (1.1.1.61), which catalyzes the conversion of 4-hydroxybutyrate into succinic semialdehyde, thereby increasing the supply of succinic acid. Similarly, the activity of the TCA cycle was promoted in Tibetan pigs, with the upregulation of the glutaconyl-CoA decarboxylase (1.3.8.1), which catalyzes the conversion of glutaconyl-CoA to grotonyl-CoA, a key reaction in both fatty acid and butyrate metabolism, significantly improving the efficiency of fatty acid utilization. In contrast, the expression of succinate dehydrogenase (1.3.5.1) and Acetoacetyl-CoA reductase (1.1.1.35) was upregulated in black pigs, enhancing the metabolic efficiency of the succinate pathway, with energy generation mainly dependent on the TCA cycle. Furthermore, the Crotonyl-CoA and butyrate metabolic pathways were significantly downregulated in black pigs compared to Tibetan pigs, indicating a weaker fatty acid metabolism in black pigs (Figure 8).

In summary, the GI microbiome of Tibetan pigs supports efficient energy metabolism by enhancing carbohydrate metabolism and short-chain fatty acid production, thereby aiding in adaptation to the low oxygen levels and high energy demands of the high-altitude environment. On the contrary, black pigs relied more on the succinate pathway and the TCA cycle, exhibiting lower flexibility in energy metabolism.

These differences in the GI microbial metabolic characteristics between pig breeds reflect the co-evolutionary relationship between the host and its intestinal flora in different ecological environments.

## 4. Discussion

Recent investigations into the GI microbiota across different segments of the mammalian digestive tract have revealed distinct microbial compositions, intricately linked to regional anatomical and physiological contexts [30,31]. Aerobic microorganisms such as various Gram-positive bacteria, fungi, and facultative anaerobes, predominantly colonize the stomach and proximal small intestine, whereas the terminal ileum shifts to a dominance of Gram-negative bacteria, and the colon and rectum are primarily inhabited by anaerobic bacteria [32,33], outnumbering aerobic microbes counterparts by 1000-fold. Our research further delineates these patterns, showing that the microbial community profiles of the stomach and jejunum are remarkably similar, characterized by a higher relative abundance of aerobic bacteria from the phyla *Bacillota* and *Pseudomonadota*. Conversely, the hindgut, comprising the cecum, colon, and rectum, exhibits a predominance of anaerobic *Bacteroidota*, underscoring a shift in microbial dynamics along the GI tract.

The comparative analysis of the GI flora in Tibetan pigs and black pigs elucidated several intriguing aspects of microbial diversity, particularly under the influence of environmental conditions like high altitude. Although the microbial diversity in the jejunum, cecum, colon, and rectum was similar between the two breeds, Tibetan pigs exhibited a notable reduction in gastric microbial diversity. This decrease is linked to the lower atmospheric oxygen levels on the Qinghai–Tibet Plateau, which, combined with the physiological occurrence of high-altitude flatus expulsion, diminishes gastric oxygen concentrations and, consequently, aerobic microbial diversity [34]. Recent multi-omics studies [35,36] have shown that it is an important tool to study adaptation and trait formation, and further research on high-altitude adaptation should also be carried out on the combined multi-omics studies of Tibetan pigs and other pig breeds.

High-altitude environments present substantial physiological challenges, potentially exacerbating gastrointestinal symptoms and systemic inflammatory responses through mechanisms such as bacterial translocation [37] and intestinal damage [38]. However, Tibetan pigs exhibit remarkable evolutionary adaptations, characterized by an enriched presence of anaerobic, short-chain fatty acid-producing probiotics throughout their gastrointestinal tract [37,39], suggesting a protective role of these bacteria against high-altitude-induced gastrointestinal distress.

This study further revealed the key role of specific probiotic genera in the adaptation of Tibetan pigs to high-altitude environments. These probiotics include *Bifidobacterium*, *Megasphaera*, *Dialister*, and *Mitsuokella*, which are not only significantly enriched in the intestines of Tibetan pigs, but also show positive intergenerational correlation, indicating that these florae enhance the stability of the host gastrointestinal tract and the ability to adapt to hypoxic conditions through synergistic effects. *Bifidobacterium* can help the host adapt to the hypoxic and hypobaric environment by enhancing the intestinal barrier function and reducing the cross-intestinal penetration of pathogens and harmful substances [40,41]. In addition, *Megasphaera*, as a typical acid-producing bacterium, can participate in the production of SCFAs, including butyrate and propionate [42]. These metabolites are not only important sources of energy for the host, but they are also significant for maintaining the health of the intestinal mucosa [43]. At the same time, the metabolic functions of *Dialister* are relatively diverse, and it participates in multiple metabolic pathways, including the production of SCFAs and the synthesis of other important metabolites [44,45]. *Mitsuokella* can produce SCFAs through fermentation processes [46], which can decompose complex carbohydrates that cannot be digested by the host [47], such as cellulose and starch, and convert them into usable energy, thereby helping the host to more effectively utilize the nutrients in food. These research results provide potential solutions for improving high-altitude gastrointestinal diseases, especially in human and veterinary medicine, the development of probiotic cocktail therapy may become an effective intervention strategy [48]. By further optimizing these probiotic combinations, theoretical support and practical guidance can be provided for alleviating common gastrointestinal dysfunction in high-altitude areas.

The importance of gut microbial metabolism in maintaining energy homeostasis and GI health is highlighted by the production of butyrate, a short-chain fatty acid critical for several physiological functions [49,50]. Butyrate is produced through the microbial fermentation of dietary fiber [51] and is crucial for maintaining the integrity of the intestinal barrier [52] and modulating inflammation within intestinal epithelial cells [53]. Beyond its local effects, butyrate is absorbed into the bloodstream [54], where it serves as an important substrate for hepatic energy metabolism [55], influencing systemic metabolic processes. Our research contributes to the growing body of evidence indicating that butyrate production is significantly enhanced in populations and species adapted to high-altitude environments, such as Tibetans, plateau pikas, and Tibetan sheep. These groups exhibit an elevated concentration of butyrate-producing bacteria in their intestines compared to their counterparts residing at lower altitudes, likely as a physiological response to the hypoxic conditions typical of high altitudes, suggesting that butyrate may provide an evolutionary advantage in these environments.

Additionally, experimental interventions using butyrate supplementation have demonstrated protective effects against intestinal damage caused by high-altitude exposure in animal models [56]. For instance, rats treated with butyrate showed significant improvements in the integrity of both the small and large intestinal barriers, emphasizing butyrate’s therapeutic potential in mitigating altitude-induced gastrointestinal dysfunctions [57]. Our study extends these findings by examining the GI microbial communities of Tibetan pigs, which are known for their inherent adaptability to high-altitude living. We identified several bacterial species, including *Clostridium* sp., *L. amylovorus*, *M. elsdenii*, *M. multacida*, *S. pleomorphus*, and uncultured strains of *Dialister* and *Megasphaera*, which showed a pronounced enrichment in butyrate metabolic pathways. This suggests a robust capacity for butyrate metabolism, likely contributing to the pigs’ physiological resilience against hypoxic stress. The consistent observation of enhanced butyrate metabolism across various high-altitude dwelling species and humans indicates a conserved adaptive mechanism [58,59,60,61] that helps maintain GI health and systemic energy balance under hypoxic conditions. The potential of butyrate supplementation as a therapeutic intervention offers promising opportunities for treating high-altitude related GI disorders [53,62]. This aligns with our broader objectives to elucidate underlying metabolic mechanisms that can be leveraged for the prognosis, prevention, and treatment of metabolic disorders and their long-term complications, especially those aggravated by or associated with environmental challenges such as high altitudes.

## 5. Conclusions

This study revealed the adaptive characteristics of Tibetan pig GI microbiota in high-altitude environments, indicating that they have enhanced their ability to survive in the harsh environment of the Qinghai–Tibet Plateau through specific microbial functions. The study found that the intestinal flora of high-altitude Tibetan pigs was significantly different from that of plain black pigs in composition and function, and key microorganisms such as *Bifidobacterium* and *Megasphaera* were enriched. These florae are significantly enriched in the degradation of complex carbon sources (such as cellulose, galactose, and fructose) and the production of short-chain fatty acids (SCFAs, such as butyrate and propionate), thereby supporting their efficient energy metabolism under hypoxic conditions. The above findings deepen our understanding of the mechanisms by which microbiota adapt to extreme environments and provide potential ideas for the development of metabolic intervention strategies based on microbial functions.

## Data Availability

The Tibetan pig and black pig bone gastrointestinal metagenomic data have been deposited in the National Center for Biotechnology Information (NCBI) Sequence Read Archive (SRA) database with the BioProject accession number PRJNA1193782.

## Supplementary Materials

The following supporting information can be downloaded at: https://www.mdpi.com/article/10.3390/ani15050753/s1, Additional file S1: Figure S1: Comparison of bacterial phyla levels between Tibetan pigs and black pigs in the colon. (A) Multiple group bar graphs of the relative abundance of *Bacillota*, *Bacteroidota*, and *Pseudomonadota*; (B) Bar graphs of the differences between *Bacillota*, *Bacteroidota*, and *Pseudomonadota* in Tibetan pigs and black pigs. Figure S2: Gene annotations for different biological pathways in the KEGG database. The vertical bar graph in the figure indicates the number of genes involved in each biological pathway. Additional file S2: Supplementary tables.xlsx: Table S1: Tibetan pig and black pig growth data; Table S2: Sequencing data information for each individual; Table S3: Top 5 relative abundance at each level; Table S4: Different KEGG corresponding microbiome information.
