# Supplementary material for: Gut Metagenome Reveals the Microbiome Signatures in Tibetan and Black Pigs
Source: Animals (Basel). 2025 Mar 6;15(5):753. doi: 10.3390/ani15050753 (PMC11899681; doi:10.3390/ani15050753)
Supplement: Supplementary file 1 [file animals-15-00753-s001.zip › Supplementary material.pdf]

Gut metagenome reveals the microbiome signatures in Tibetan and Black Pigs

A

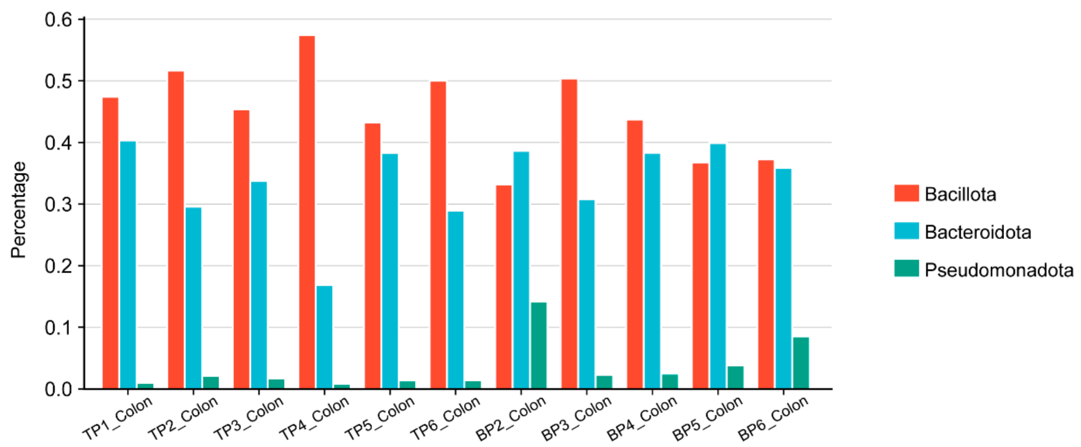

B

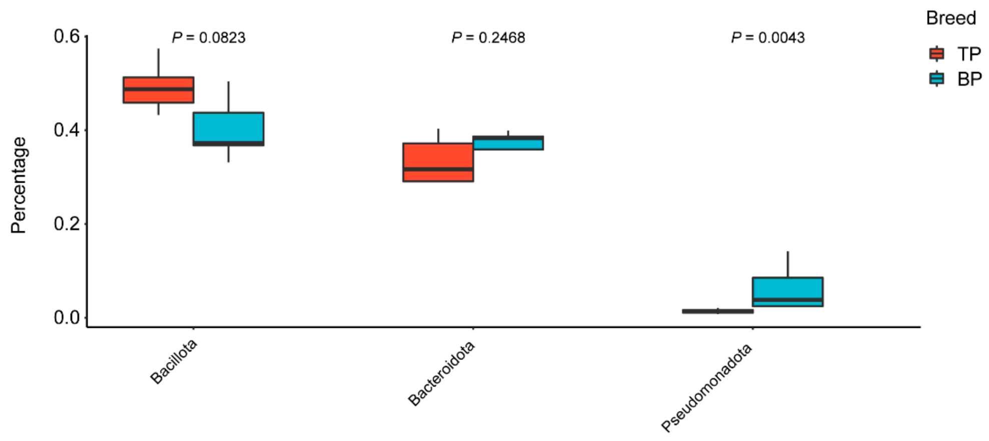

Figure S1. Comparison of bacterial phyla levels between Tibetan pigs and black pigs in the colon. (A) Multiple group bar graphs of the relative abundance of *Bacillota*, *Bacteroidota*, and *Pseudomonadota*; (B) Bar graphs of the differences between *Bacillota*, *Bacteroidota*, and *Pseudomonadota* in Tibetan pigs and black pigs.

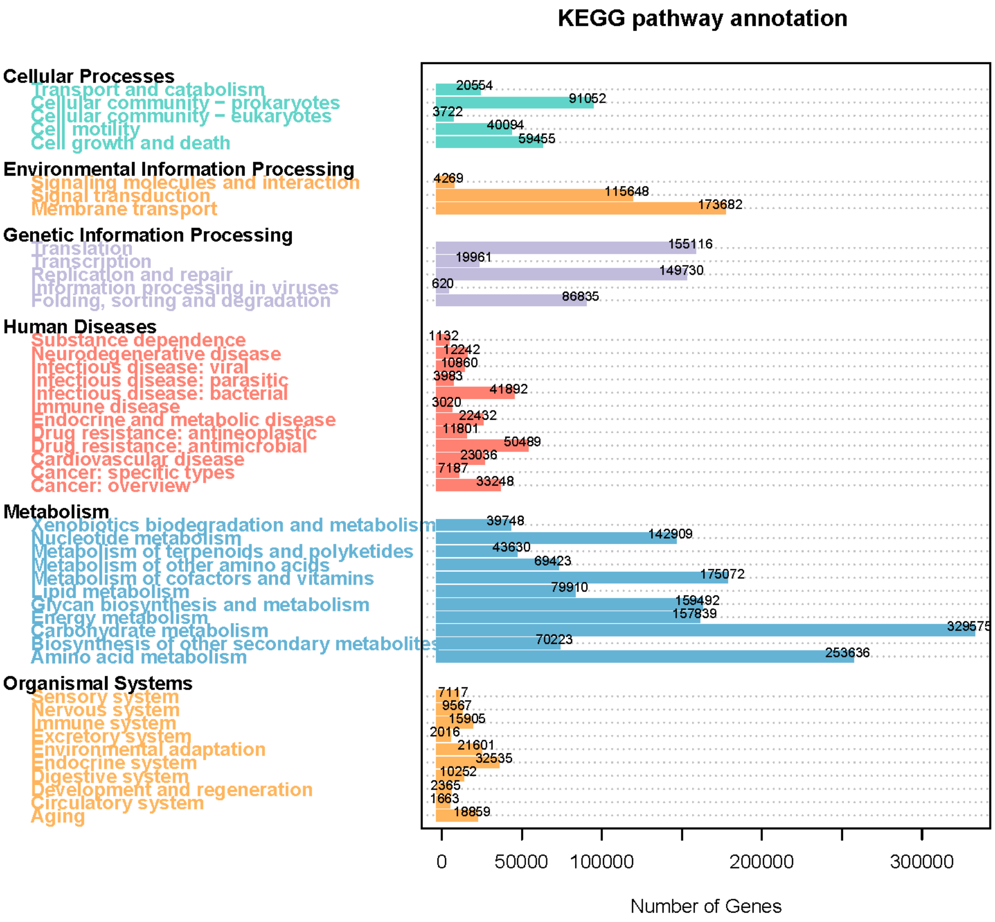

Figure S2. Gene annotations for different biological pathways in the KEGG database. The vertical bar graph in the figure indicates the number of genes involved in each biological pathway.

Supplementary information on animal management and feeding conditions

During the 300-day stable feeding period, all Tibetan pigs and black pigs were raised under standardized conditions. The following are the details:

1. Feeding environment

Each pig was provided with a feeding space of 3.0 m^2/head to ensure sufficient room for activities.

Temperature control: Black pigs were raised at low altitudes (461 m above sea level), with a naturally changing ambient temperature ranging from 15-25°C, and equipped with sunshade and ventilation equipment to cope with hot weather; Tibetan pigs were raised at high altitudes (3750 m above sea level), with an ambient temperature of 5-15°C, and provided with insulation facilities to ensure the comfort of pigs in cold weather.

Bedding: Straw was provided as bedding during feeding and was changed regularly to keep the pens clean and dry.

2. Diet composition

Feeding a uniformly formulated basic diet, the diet composition is as follows:

Energy feed: corn accounts for about 60%;

Protein feed: soybean meal accounts for about 25%;

Crude fiber feed: bran and silage account for about 10%;

Mineral and vitamin additives: account for about 5%.

Drinking water: All pigs can drink clean water freely, and the drinking water device is cleaned daily to ensure hygiene.

Feeding frequency: Feed twice a day, at 8 am and 5 pm.

3. Feeding management

During the entire feeding cycle, the health status of the experimental pigs was checked regularly to ensure that they were not affected by disease or stress.

No antibiotics or hormone drugs were used for any pigs.
